# Effects of brushing with caffeinated toothpaste on neurocognitive function of the central nervous system: a randomized placebo-controlled clinical trial

**DOI:** 10.3389/froh.2025.1470531

**Published:** 2025-05-15

**Authors:** Kiarash Zare, Mahva Talaei, Amir Hesam Pahlevani, Fahimeh Rezazadeh, Kiana Zare, Masumeh Akbaryari, Mohammad Mehdi Naghizadeh, Mojtaba Heydari, Mohsen Goharinia

**Affiliations:** ^1^ Neuroscience Research Group, Student Research Committee, Fasa University of Medical Sciences, Fasa, Iran; ^2^ Department of Oral and Maxillofacial Medicine, School of Dentistry, Shiraz University of Medical Sciences, Shiraz, Iran; ^3^ Noncommunicable Diseases Research Center, Fasa University of Medical Sciences, Fasa, Iran; ^4^ Poostchi Ophthalmology Research Center, Shiraz University of Medical Sciences, Shiraz, Iran; ^5^ Research Center for Traditional Medicine and History of Medicine, Department of Persian Medicine, School of Medicine, Shiraz University of Medical Sciences, Shiraz, Iran; ^6^ Department of Pharmacology, School of Medicine, Fasa University of Medical Sciences, Fasa, Iran

**Keywords:** caffeine, brushing, toothpaste, cognitive, memory, reaction, stimulant, drug delivery

## Abstract

**Background:**

This randomized placebo-controlled clinical trial aimed to evaluate the effect of brushing with caffeinated toothpaste on neurocognitive function of the central nervous system.

**Methods:**

Eighty healthy individuals were randomly assigned to four groups: oral caffeine capsules (100 mg caffeine) as the control, brushing with caffeinated toothpaste (100 mg caffeine) for 2, 3, and 4 min. Cognitive and motor responses were assessed using selective processing speed assessment (Stroop test), short-term memory test, selective attention capacity assessment, and hand-eye coordination test before and after intervention at 10, 30, and 60 min intervals.

**Results:**

Brushing with caffeinated toothpaste was as effective as oral caffeine intake in improving selective attention capacity, selective processing speed, short-term memory, and hand-eye coordination. Despite the higher improvement in the longest duration brushing group in most of the outcomes, the difference did not reach the statistical significance among study groups.

**Conclusion:**

Brushing with caffeinated toothpaste appears to be as effective as oral intake of caffeine in enhance cognitive and motor functions.

**Clinical Trial Registration:**

https://irct.behdasht.gov.ir/trial/71213, identifier (IRCT20230318057752N2).

## 1 Introduction

Caffeine, a naturally occurring stimulant, is ubiquitous in our daily lives and is primarily sourced from plants such as coffee beans, tea leaves, and cocoa (1, 2). With a rich historical tapestry dating back centuries, caffeine has played a pivotal role in human societies, transcending cultural and geographical boundaries (3, 4). Renowned for its stimulating properties, caffeine has become an integral component of many beverages and products, making its way into various aspects of our routine (5). As a psychoactive substance, its impact on the central nervous system (CNS) has intrigued researchers and scientists, leading to extensive investigations into its physiological and cognitive effects (6).

Caffeine's influence on the CNS is profound and well-documented (7). As an adenosine receptor antagonist, caffeine exerts its effects by blocking adenosine, a neurotransmitter responsible for promoting sleep and relaxation (8). By inhibiting adenosine, caffeine enhances the release of other neurotransmitters like dopamine and norepinephrine, leading to heightened alertness and improved cognitive function (9, 10). The well-established role of caffeine in mitigating fatigue and enhancing mental acuity has prompted numerous studies to delve deeper into its potential benefits and explore novel avenues for its delivery.

Beyond traditional modes of caffeine consumption, such as drinking coffee or tea, researchers have explored alternative methods of delivery to unlock the full spectrum of its cognitive and motor-boosting potential (11). Intriguingly, studies have investigated diverse formulations, including caffeinated chewing gums, transdermal patches, and even intranasal administration (12, 13). These explorations into innovative delivery mechanisms aim to optimize the efficacy of caffeine, opening avenues for its application in cognitive enhancement.

Motivated by the multifaceted nature of caffeine's impact on the CNS and the ongoing quest for innovative delivery methods, this manuscript embarks on a pioneering investigation. The primary aim of this randomized clinical trial was to evaluate whether brushing with caffeinated toothpaste can induce cognitive and motor responses within the central nervous system comparable to those observed with traditional modes of caffeine ingestion in different time intervals. The secondary aim was to evaluate the effect of different durations of brushing on the enhancing neurocognitive function. While the cognitive and motor-enhancing effects of caffeine are well-established, the novelty of our study lies in evaluating an alternative mode of caffeine delivery—via brushing with caffeinated toothpaste.

## 2 Materials and methods

### 2.1 Trial design

This study was a randomized placebo-controlled clinical trial using the parallel groups. We evaluated the effect of absorption of caffeine through brushing with caffeinated toothpaste on the cognitive and motor responses of the central nervous system.

During a public call, students were invited to participate in the study. Volunteer students were referred to the virtual education center of Fasa University of Medical Sciences, Explanations about the study were given to them, and informed written consent was obtained from them.

This study was carried out in the Comprehensive Virtual Center, affiliated with Fasa University of Medical Sciences, Fasa, Iran, from September 2023 to October 2023.

### 2.2 Preparation of the intervention and control toothpastes and capsules

The caffeinated toothpaste utilized in the study, YUZ Energy Boost Caffeine Toothpaste, was sourced from Dr. Kaschny Healthcare GmbH Company in Germany, with each cubic centimeter (CC) containing 100 mg of caffeine. This dose was selected based on the previous studies (14). For the control group, non-caffeinated toothpaste (Colgate-Palmolive Company, USA) was employed. Additionally, caffeinated capsules, each containing 100 mg of caffeine, were procured from Jalinous Pharmaceutical Company in Iran. Placebo capsules were meticulously crafted using starch powder in lieu of caffeine, replicating the appearance of the active capsules. Notably, all capsules were packaged similarly to maintain consistency.

### 2.3 Eligibility criteria

This study sought participation from individuals meeting specific inclusion criteria. Inclusion criteria comprised those in good health and individuals who willingly provided informed consent to engage in the study.

On the other hand, exclusion criteria were established to screen out individuals who might introduce confounding variables. Participants demonstrating any reaction or sensitivity to caffeine were excluded, as were those addicted to drugs, alcohol, or tobacco. Additionally, individuals who had consumed coffee, painkillers containing caffeine, nerve agents, tea, or other potentially influencing drinks within 48 h before the study were not considered eligible. Furthermore, exclusion criteria encompassed individuals with cognitive disorders, mental disorders, cardiovascular diseases, neurological disorders, thyroid disorders, or those afflicted with mouth or mucous membrane diseases.

### 2.4 Sample size calculation

According to the study by Skinner et al. (15), the median performance improved by about 4% (interquartile range = 9%), so the standard deviation (SD) was estimated at 4.5. To achieve a 4% significance, change in performance function, with SD = 4.5, The sample size were calculated to be 17 participants in each group (α = 0.05, β = 0.2) using the below formula. Considering the risk of drop out 20 participants enrolled in each group.

$$n=\frac{2{\left(Z_{\left(1-\alpha /2\right)}+Z_{1}-\beta \right)}^{2}S^{2}}{d^{2}}$$

### 2.5 Study groups

**Figure 1:**
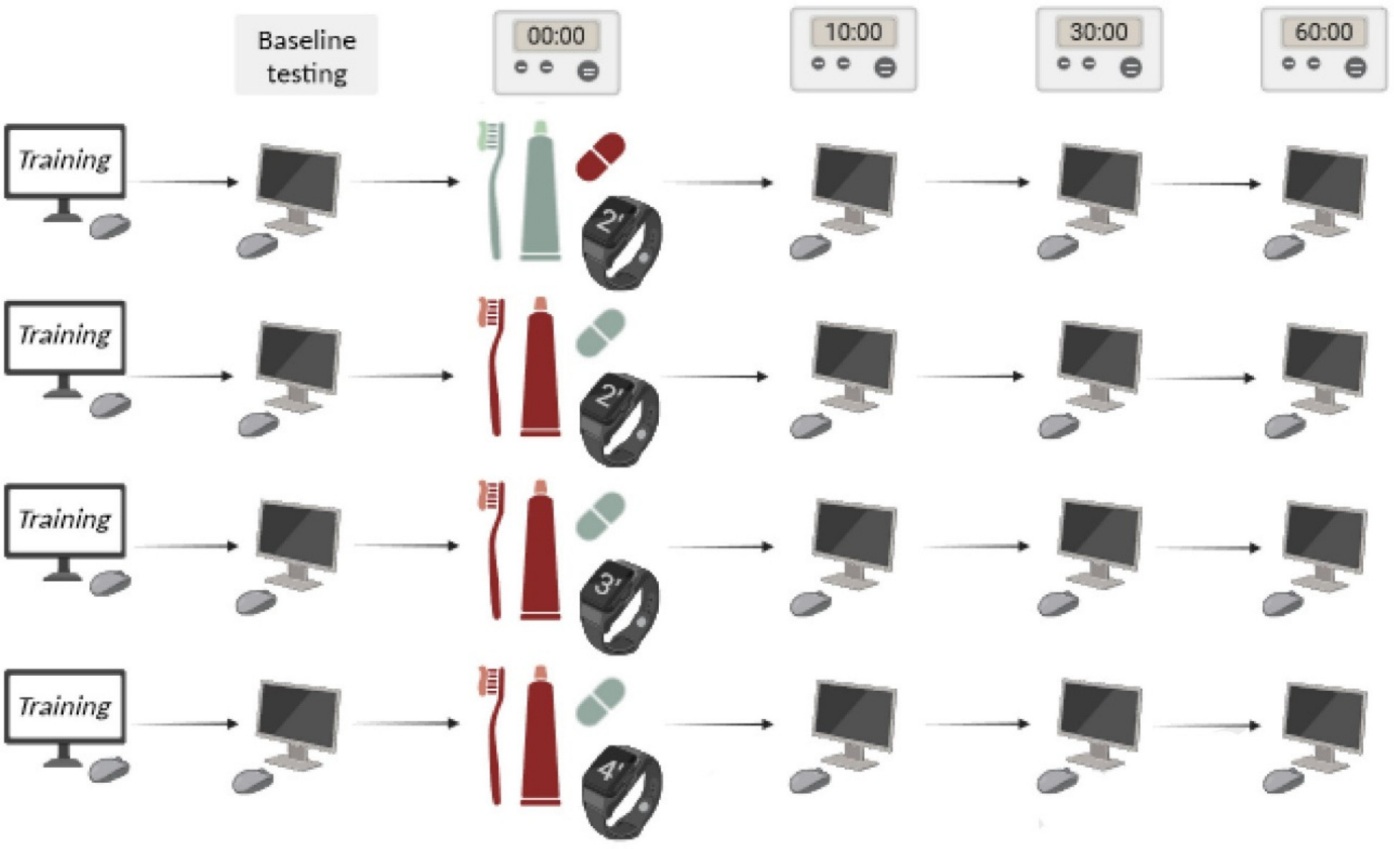
Workflow of study: the rows represent the study groups. Pale green means placebo and red means the presence of caffeine (Created with BioRender.com). All study participants received training course on neuro-cognitive tests and performed the test 1 time before and 3 times after the intervention (in 10, 30, and 60 min intervals).

This study was designed for four groups, which included: (1). The recipients of a 100 mg caffeine capsule and toothbrush with regular toothpaste in 2 min (control group), (2). The recipients of the placebo capsule and toothbrush with caffeinated toothpaste 100 mg in 2 min, (3). The recipients of the placebo capsule and toothbrush with caffeinated toothpaste 100 mg in 3 min and (4). The recipients received the placebo capsule and toothbrush with caffeinated toothpaste at 100 mg in 4 min (Figure 1).

### 2.6 Randomization

Participant randomization was conducted utilizing a permutation strategy with four distinct blocks—Groups A, B, C, and D—each representing a combination of capsules and toothpaste with and varying brushing durations (Figure 1).

To achieve randomization, permutations of these blocks were generated, and the random sequence was executed using the RAND function in MS Excel software. Subsequently, participants were assigned in accordance with the block-sequence order. The capsules and toothpaste were identical in shape and packaging. This measure aimed to prevent both researchers and participants from discerning the content (i.e., one capsule, toothpaste, and toothbrush) of each individual's package. The person responsible for drug packaging alone possessed knowledge of the envelope numbers, maintaining the confidentiality of the allocation.

### 2.7 Participants' enrollment

After explaining the study to the participants, once before starting the study, all the computer tests were shown to them in a visual form, and then once, the participants did all the tests for training. Then they performed the computer tests once, and the results were recorded as the baseline. Then, people were given packages containing toothpaste, capsules, and toothbrushes, and they were asked to brush their teeth according to the instructions on the package and then eat the capsules. All brushing sessions were conducted in person at the study site under direct supervision to ensure adherence to the protocol. Participants followed the brushing instructions on-site, and their brushing duration was monitored to maintain accuracy in group allocation. To ensure consistency in the amount of toothpaste used, all brushing sessions were supervised to confirm that participants used the full 1 cc of toothpaste as instructed. Ten, thirty, and sixty minutes after brushing and taking the capsule, the participants performed computer tests. During this time, the participants were asked not to eat anything and to be present at the study site.

The study took about 1:30 h for each participant. The participants were called to the study place between 3:00 p.m. and 6:00 p.m. We tried to make the study conditions as similar as possible for the participants. For this reason, all computer systems were the same when using the facilities of the Fasa University of Medical Sciences test center. Also, the mouse used in all systems was similar.

### 2.8 Neuro-cognitive tests

**Figure 2:**
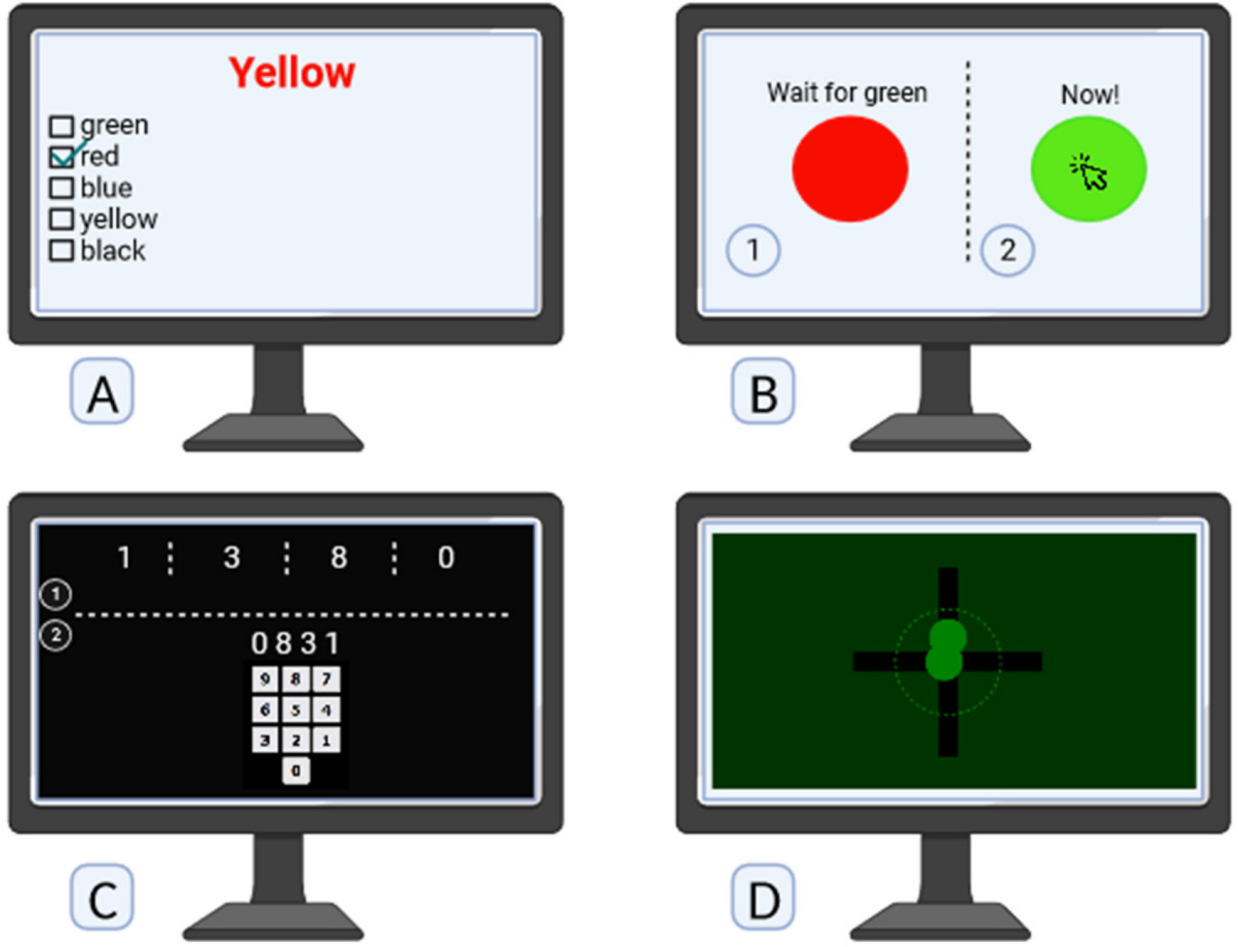
Neuro-cognitive computer tests: **(A)** selective attention capacity by multiple-choice stroop test score. **(B)** Selective processing speed by reaction time. **(C)** Short term memory test. **(D)** Hand eye coordination test.

In this study, the participants were evaluated using four online computer-based neuro-cognitive tests (Figure 2). We utilized psychological tests available on the AREALME platform (arealme.com), a renowned site for engaging and innovative online quizzes, tests, and games, which originated at Singapore Management University and has been previously employed in studies (16). The Stroop test were considered as the primary outcome and the other tests as secondary outcomes. The details of the online computer tests are as follows:

#### 2.8.1 Selective attention capacity assessment

Selective attention capacity was assessed using the Stroop test, a widely recognized cognitive evaluation tool (17). The multiple-choice version of Stroop test employed a list of words where the color of the word and its meaning were intentionally incongruent. For instance, the word “yellow” might be written in red ink. Participants were tasked to select the color of the ink used to print the words from multiple choices, requiring them to selectively attend to the color information while suppressing the automatic processing of the word's meaning. The Stroop test is designed to measure the efficiency of selective attention and inhibitory control, offering valuable insights into cognitive processes and the ability to focus on specific aspects of stimuli while ignoring conflicting information (https://psycho-tests.com/test/stroop-test).

#### 2.8.2 Selective processing speed assessment

Selective processing speed was evaluated using a reaction time paradigm (18). Participants engaged in a task where a red circle initially presented would abruptly transition to green after a brief duration. The objective was for participants to swiftly click on the green circle upon its appearance. This process was iterated five times, and the average reaction speed of the participants was subsequently recorded. The test aimed to measure the speed and efficiency with which individuals could selectively process and respond to a specific visual stimulus, in this case, the color change from red to green. The test provides 3 values of total responses, correct responses and mean reaction time for response (https://www.arealme.com/reaction-test/en).

#### 2.8.3 Short-term memory test

The Short-Term Memory Test involved the presentation of randomly generated sets of numbers on a screen (19). Following the display, participants were required to enter the sequence of numbers in reverse order. This test specifically targeted short-term memory capabilities, assessing participants' ability to temporarily retain and manipulate information (https://www.arealme.com/brain-memory-game/en).

#### 2.8.4 Hand-eye coordination test

The Hand-Eye Coordination Test focuses on the synchronized control of eye movement with hand reactions. Each round of the test involves the presence of two balls, two axes, and a white dashed circle on the screen. The balls move along the axes, and the objective for participants is to skillfully manipulate their hand movements to bring the moving balls to a stop within the confines of the white dashed circle. This test serves as a dynamic assessment of the integration between visual perception and manual dexterity, offering insights into the precision and effectiveness of hand-eye coordination (https://www.arealme.com/eye-hand-coordination-test/en).

### 2.9 Ethical issues

The trial followed the Declaration of Helsinki (1989 revision) and was also reviewed, approved, and monitored by the ethics committee of Fasa University of Medical Sciences (License Number IR.FUMS.REC.1402.042). The trial was registered with the Iranian Registry of Clinical Trials with the following code: IRCT20230318057752N2. All the participants signed an informed consent form prior to enrollment in the study.

### 2.10 Statistical methods

All data collected for the study underwent comprehensive analysis using the Statistical Package for the Social Sciences (SPSS software, Version 27). The statistical analyses encompassed Per Protocol analysis in both descriptive and analytical approaches to provide a thorough understanding of the observed outcomes. Descriptive statistics, including means, standard deviations, frequencies, and percentages, were employed to summarize and characterize the key features of the datasets. For inferential analysis, inferential statistical tests were applied to examine potential associations, differences, or trends in the data. An analysis of variance (ANOVA) test was specifically employed for comparing means across multiple groups. *post hoc* tests, such as Bonferroni test, were further employed to pinpoint specific group differences if the ANOVA results indicated overall significance. Dunnett's test was employed for multiple comparisons among groups. The significance level was set at *p* < 0.05.

## 3 Result

### 3.1 Study flow and basic characteristics

**Figure 3:**
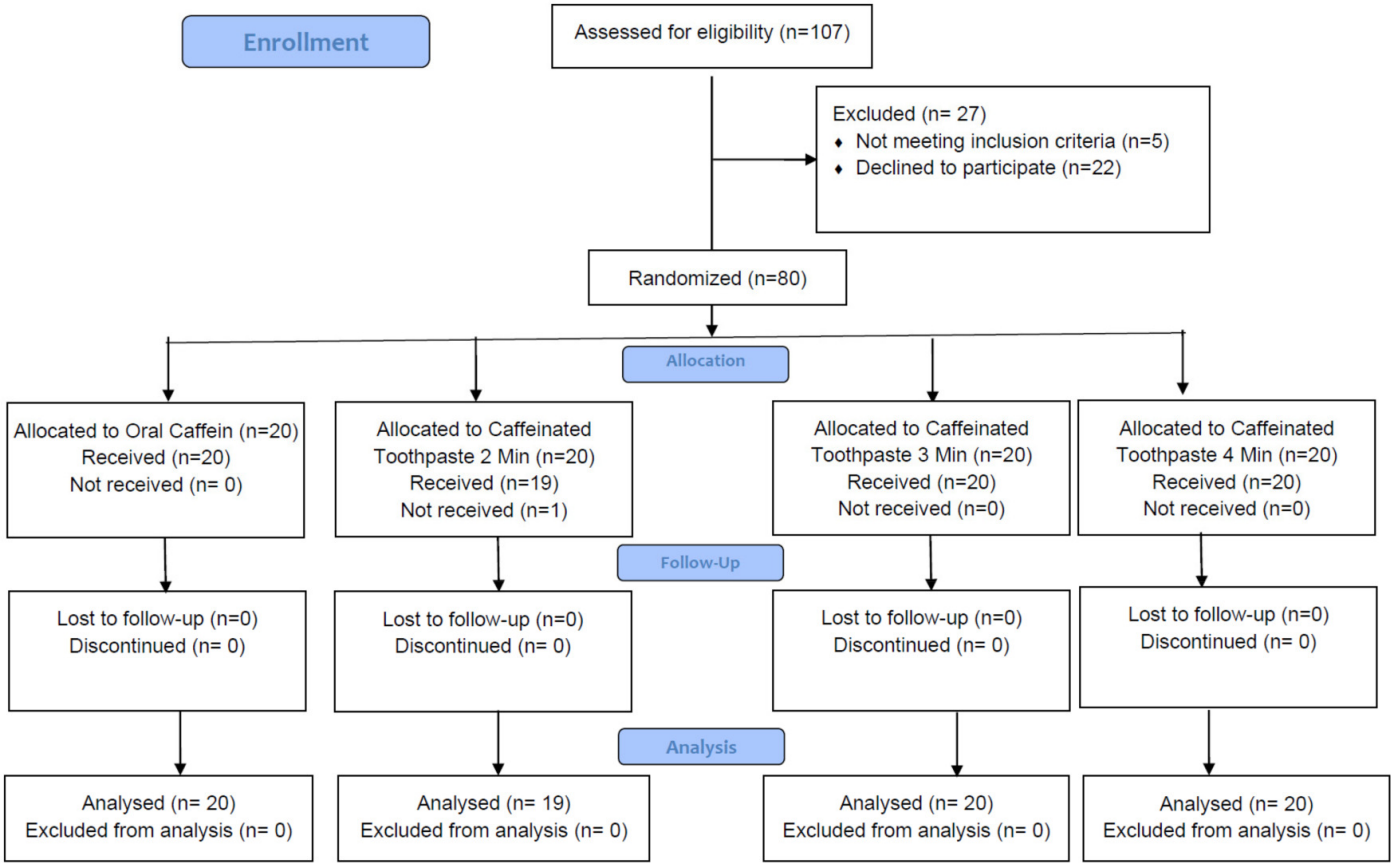
Consort flow diagram of the participants enrollment, allocation and follow-up.

Of the 107 participants assessed for eligibility, 27 were excluded due to not meeting inclusion criteria (*n* = 5) or declining to participate (*n* = 22). Eighty eligible participants were randomized into four groups: oral caffeine (*n* = 10), brushing with caffeinated toothpaste for 2 min (*n* = 10), 3 min (*n* = 10), and 4 min (*n* = 10). All participants in the oral caffeine, 3 min, and 4 min brushing groups received their allocated interventions, while one participant in the 2 min brushing group did not. A total of 39 participants received and completed the intervention, with all participants analyzed in their respective groups (Figure 3).

The basic characteristics of included participants are summarized in Table 1. Totally, 36 men and 43 women participated in this study with the mean age of 22.38 ± 2.31.

**Table 1:** Baseline characteristics of the study participants.

Study groups	Gender male/female	Age (years) mean ± SD	Weight (kg) mean ± SD
Oral caffeine capsule (100 mg)	8/12	22.55 ± 1.76	77.55 ± 32.62
Brushing with caffeinated toothpaste (2 min)	5/14	21.63 ± 2.54	68.79 ± 25.53
Brushing with caffeinated toothpaste (3 min)	8/12	22.10 ± 2.29	64.90 ± 9.99
Brushing with caffeinated toothpaste (4 min)	15/5	23.20 ± 2.44	72.30 ± 11.23
Total	36/43	22.38 ± 2.31	70.91 ± 22.07

### 3.2 Stroop test

**Figure 4:**
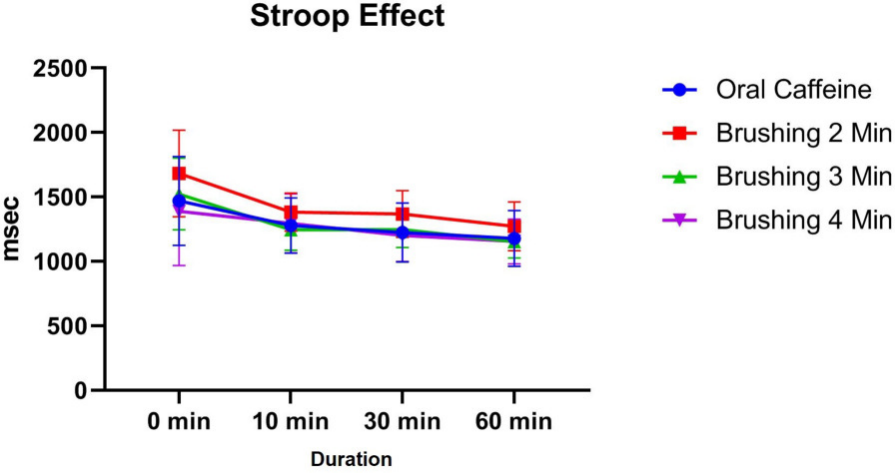
The trend of changes in cognitive performance based on Stroop test results for different study groups at different time intervals (before and 10, 30 and 60 min after intervention).

The Stroop test results, as the primary outcome, demonstrated comparable baseline performance across the groups, with the oral caffeine group showing a mean Stroop effect of 1469.35 ± 345.21 ms, while the 4 min brushing group had the lowest mean at 1388.94 ± 422.07 ms. At 10 min post-intervention, the mean Stroop effect improved across all groups, with the 3 min brushing group showing the most improvement (1245.45 ± 158.52 ms), though no significant differences were observed between the oral caffeine group and the brushing groups (*p* > 0.05). By 30 min, further improvements were noted, particularly in the 4 min brushing group (1200.05 ± 200.37 ms), but these differences remained non-significant compared to oral caffeine (1223.9 ± 230.05 ms; *p* > 0.05). At 60 min, the 3 min (1152.2 ± 126.66 ms) and 4 min (1154.6 ± 173.58 ms) brushing groups showed slightly better performance than the oral caffeine group (1177.85 ± 217.16 ms), but no statistically significant differences were detected (*p* > 0.05) (Figure 4).

### 3.3 Reaction time

The reaction time results showed no significant differences across groups at baseline (0 min), with the oral caffeine group having a mean reaction time of 283.1 ± 42.58 ms and the brushing groups ranging from 276.95 ± 33.58 ms (3 min) to 292.37 ± 28.3 ms (2 min; *p* > 0.05). At 10 min post-intervention, mean reaction times improved slightly across all groups, with the oral caffeine group at 274.7 ± 36.92 ms and the brushing groups ranging from 271.05 ± 46.48 ms (3 min) to 292.05 ± 46.83 ms (2 min), with no significant differences observed (*p* > 0.05).

**Figure 5:**
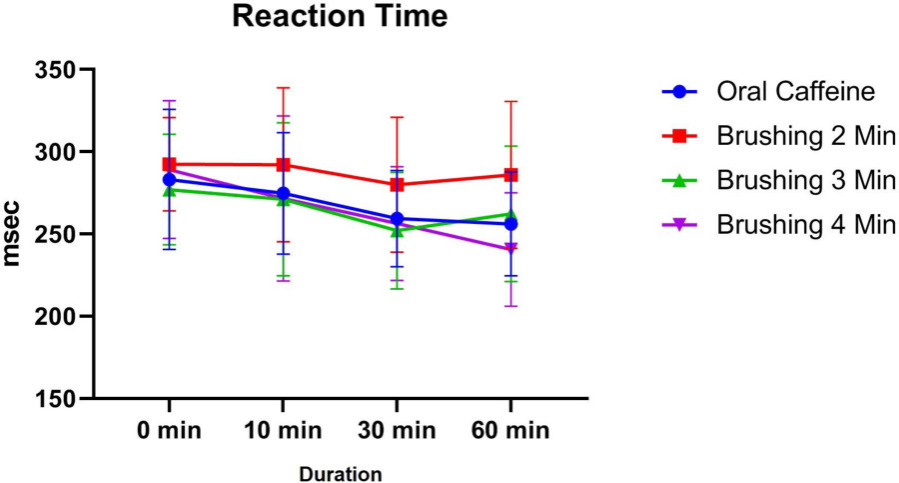
The trend of changes in cognitive performance based on reaction time for different study groups at different time intervals (before and 10, 30 and 60 min after intervention).

By 30 min, the mean reaction time was lowest in the 3 min brushing group (252 ± 35.47 ms) compared to the oral caffeine group (259.4 ± 29.26 ms), but the differences between groups remained non-significant (*p* > 0.05). At 60 min, the 4 min brushing group exhibited the best performance (240.55 ± 34.52 ms), followed by the oral caffeine group (256.15 ± 31.61 ms). A statistically significant difference was observed between the oral caffeine group and the 2 min brushing group at this time point (−29.8 ms; *p* = 0.0443). However, all other comparisons showed no significant differences (*p* > 0.05) (Figure 5).

### 3.4 Short term memory

The short-term memory test results revealed no significant differences across groups at baseline (0 min), with the oral caffeine group achieving a mean score of 79 ± 12.1 and the brushing groups ranging from 72.5 ± 11.18 (4 min) to 73.5 ± 15.99 (3 min; *p* > 0.05). At 10 min post-intervention, the oral caffeine group showed the highest mean score (85.5 ± 9.45), while the 2 min brushing group exhibited a significantly lower score (74.47 ± 10.39) compared to oral caffeine (*p* = 0.0095). However, no significant differences were observed between the oral caffeine group and the 3 min (82 ± 12.4) or 4 min (79 ± 11.65) brushing groups at this time point (*p* > 0.05).

**Figure 6:**
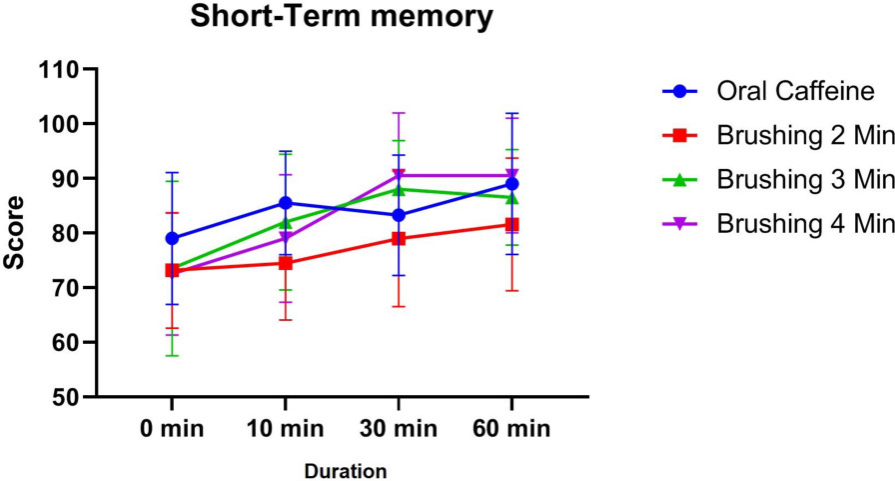
The trend of changes in cognitive performance based on short term memory results for different study groups at different time intervals (before and 10, 30 and 60 min after intervention).

At 30 min, the highest performance was observed in the 4 min brushing group (90.5 ± 11.46), followed by the 3 min group (88 ± 8.94) and the oral caffeine group (83.25 ± 11.04), but the differences were not statistically significant (*p* > 0.05). By 60 min, all groups showed improved performance, with the 4 min brushing group maintaining the highest mean score (90.5 ± 10.5), while the oral caffeine group and the 3 min brushing group achieved similar results (89 ± 12.94 and 86.5 ± 8.75, respectively). No significant differences were observed between the oral caffeine and brushing groups at 60 min (*p* > 0.05) (Figure 6).

### 3.5 Hand eye coordination

At baseline (0 min), the oral caffeine group demonstrated the highest mean performance (56.9 ± 12.53), with brushing groups ranging from 44.37 ± 19.41 (2 min) to 54.8 ± 15.57 (4 min). At 10 min, all groups improved, with the 4 min brushing group achieving the highest mean score (61.9 ± 19.85) and the oral caffeine group closely following (59.85 ± 12.22). The brushing groups for 2 and 3 min scored 51.84 ± 19.32 and 54.25 ± 16.32, respectively. By 30 min, the oral caffeine group and the 4 min brushing group remained comparable (64.35 ± 15.18 and 61.85 ± 17.65, respectively), with no significant differences across groups at this time point (*p* > 0.05).

**Figure 7:**
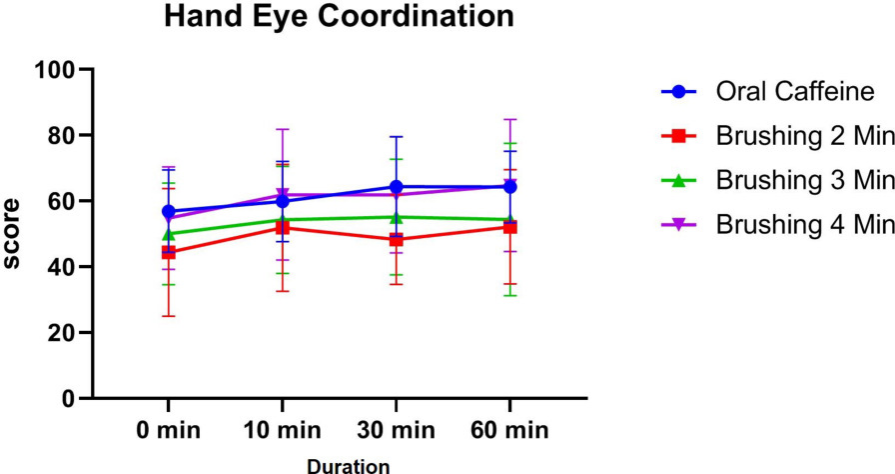
The trend of changes in hand eye coordination test results for different study groups at different time intervals (before and 10, 30 and 60 min after intervention).

At 60 min, the 4 min brushing group showed the highest performance (64.7 ± 20.06), followed closely by the oral caffeine group (64.25 ± 10.88). The 2 min and 3 min brushing groups had lower scores (52.16 ± 17.4 and 54.35 ± 23.15, respectively). While the column factor was significant overall (*p* < 0.0001), reflecting changes over time, no individual group differences reached statistical significance compared to oral caffeine (*p* > 0.05) (Figure 7).

### 3.6 Peak performance times

The results demonstrated variations in performance enhancement across different time points (10, 30, and 60 min) for the cognitive and motor function tests. The most significant improvements in selective processing speed, as measured by the Stroop test, were observed at 60 min in all groups, with oral caffeine achieving the shortest reaction time (mean = 1177.85 ms) compared to brushing groups. Similarly, for reaction time tests, participants displayed optimal performance at 60 min, with the 4 min brushing group showing a mean time of 240.55 ms, closely aligning with the oral caffeine group (mean = 256.15 ms). In short-term memory tests, the highest scores were observed at 60 min, where the 4 min brushing group (mean = 90.5) surpassed other brushing groups and was comparable to oral caffeine (mean = 89). For hand-eye coordination, the peak performance was noted at 60 min in the 4 min brushing group (mean = 64.7) and the oral caffeine group (mean = 64.25), indicating similar efficacy. These findings suggest that cognitive and motor improvements following caffeinated interventions reach their peak around 60 min, regardless of delivery method.

### 3.7 Adverse effects

No serious adverse event was reported in any participant. Only participants who received caffeinated toothpaste complained the bitter taste of caffeine after brushing.

## 4 Discussion

The study investigated the impact of brushing with caffeinated toothpaste on neuro-cognitive and motor responses. The control group, which received oral caffeine, was compared with the groups brushed with caffeinated toothpaste for 2, 3, and 4 min, respectively. The measured outcomes included Selective Processing Speed Assessment (Stroop test), Short-Term Memory Test, Hand-Eye Coordination Test, and Selective Attention Capacity Assessment. The findings revealed that all groups exhibited significant improvements in neurocognitive functions, indicating the efficacy of both oral caffeine intake and brushing with caffeinated toothpaste. Moreover, brushing with caffeinated toothpaste demonstrated better effects with longer brushing durations.

The observed effects of brushing with caffeinated toothpaste on cognitive and motor responses may arise from a combination of multifaceted mechanisms (20). Caffeine, whether consumed orally or delivered via toothpaste, acts as a potent stimulant for the central nervous system (21). By blocking adenosine receptors, caffeine heightens alertness and arousal, potentially leading to enhancements in various cognitive functions, including attention, memory, and processing speed (22–24). Additionally, caffeine's influence on neurotransmitter systems implicated in cognition, such as dopamine and acetylcholine, may further amplify its cognitive-enhancing effects (7, 8).

Caffeine can be delivered through various methods, including oral ingestion, transdermal application, and mucosal absorption, each offering unique benefits for different needs and circumstances (11). Oral ingestion, such as through coffee, tea, or energy drinks, is the most common and convenient method, providing a quick systemic effect via gastrointestinal absorption. Transdermal patches provide a slower, steady release of caffeine, making them ideal for prolonged use and minimizing gastrointestinal discomfort (25). Mucosal absorption, found in lozenges or gum, allows for rapid absorption directly into the bloodstream through the oral cavity, bypassing the digestive system for a faster onset of effects (26). These varied delivery methods highlight the flexibility of caffeine as a stimulant in different delivery method, as seen in our study on brushing with caffeinated toothpaste.

Our study, which investigated the effects of caffeine delivery by brushing with caffeinated toothpaste on cognitive and motor functions, corresponds with several other inquiries exploring the impact of caffeine on various performance aspects. Pirmohammadi et al. (27) examined the effects of early absorption sources of caffeine, including caffeinated gum and coffee mouth rinsing, on female table tennis players (27). They discovered that both caffeinated gum and coffee mouth rinsing significantly improved agility and reduced errors in cognitive tests compared to a placebo. These findings resonate with our observation that both oral and toothpaste caffeine enhanced cognitive functions such as selective processing speed and short-term memory. This suggests a potential overlap in the mechanisms underlying the cognitive effects of caffeine across different delivery methods.

Similarly, Wu et al. (28) explored the effects of caffeine supplementation on elite e-sports players' cognitive abilities and shooting performance (28). They noted significant improvements in reaction times and shooting performance following caffeine supplementation. While their study focused on a different population and delivery method, the observed enhancements in cognitive and motor functions parallel our findings of improved cognitive functions and less significant improvements in motor functions with oral and toothpaste caffeine.

Additionally, Moradi et al. (29) investigated the effectiveness of caffeinated chewing gum in ameliorating cognitive functions affected by sleep deprivation (29). They found that a higher dose of caffeine (300 mg) led to greater enhancement of cognitive functions compared to a lower dose (200 mg). This finding resonates with our observation that brushing with toothpaste containing caffeine (100 mg) led to improvements in cognitive functions, suggesting a potential dose-dependent effect of caffeine on cognitive performance. Our observed more significant effect of longer duration brushing with caffeinated toothpaste support the dose dependent effect of caffeine.

The finding that the 2 min brushing group had the highest Stroop test scores but the lowest short-term memory and hand-eye coordination scores is indeed worth discussing. One possible explanation could be the differential absorption dynamics of caffeine through the oral mucosa. Shorter brushing durations may lead to rapid but limited absorption, which could provide an early boost in cognitive tasks involving selective attention (such as the Stroop test) but may not sustain the effects required for tasks like memory recall and motor coordination. Additionally, individual variability in caffeine metabolism and receptor sensitivity could contribute to these variations. It is also possible that brushing for 2 min allowed sufficient absorption to enhance selective attention but did not reach the threshold needed for improvements in more complex neurocognitive and motor tasks.

Despite the robustness of our study design, several limitations should be noted. One limitation pertains to the homogeneity of our study population, which consisted solely of healthy young individuals meeting specific inclusion criteria. Consequently, caution is warranted when generalizing our findings to broader populations, such as individuals with underlying medical conditions or diverse demographic backgrounds. Additionally, the relatively short duration of our study may have hindered the detection of long-term effects associated with absorption of caffeine through brushing with caffeinated toothpaste. Future research with extended follow-up periods could provide valuable insights into the sustained impact of this intervention. Moreover, while efforts were made to standardize the brushing procedure across participants, variations in brushing technique or compliance could have influenced the absorption of caffeine and subsequent cognitive and motor responses. Furthermore, the utilization of online computer-based neuro-cognitive tests, while convenient, may not fully capture the intricacies of real-world cognitive and motor functions. Lastly, the relatively small sample size may have limited the statistical power of our analyses, potentially obscuring subtle effects of the intervention. Nonetheless, our study contributes significant insights into the potential cognitive and motor effects of absorption of caffeine through brushing with caffeinated toothpaste, serving as a foundation for further investigations in this area.

While caffeine can offer various benefits, excessive consumption can lead to several negative effects. These may include increased heart rate, elevated blood pressure, anxiety, insomnia, and digestive issues (30). Overuse can also lead to dependence, causing withdrawal symptoms such as headaches, fatigue, and irritability. In vulnerable individuals, such as those with heart conditions or pregnant women, high caffeine intake can pose greater risks, making moderation essential to avoid potential harm (31).

In conclusion, our randomized placebo-controlled clinical trial demonstrated no significant difference in the study outcomes between different study groups. This shows that brushing with caffeinated toothpaste appears to be as effective as oral intake of caffeine in enhance cognitive and motor functions. Our findings suggest that this novel delivery method holds promise for enhancing cognitive functions, such as selective attention and short-term memory, while potentially influencing hand-eye coordination. Future research endeavors should aim to replicate and expand upon our findings, exploring the long-term effects and optimal dosages of caffeine delivered through this route. Overall, our study contributes to the growing body of evidence supporting the use of local oral absorption of caffeine as a potential strategy for enhancing cognitive and motor functions, with implications for various domains, including education, sports, and occupational performance.

## Data Availability

The data of this study are available from the corresponding author upon reasonable request.
