# Supplementary material for: Effects of brushing with caffeinated toothpaste on neurocognitive function of the central nervous system: a randomized placebo-controlled clinical trial
Source: Front Oral Health. 2025 May 15;6:1470531. doi: 10.3389/froh.2025.1470531 (PMC12119480; doi:10.3389/froh.2025.1470531)
Supplement: Supplementary file 1 [file Table1.docx]

**Hand Eye Coordination**

| Two-way ANOVA | Ordinary |  |  |  |  |  |  |  |  |  |  |  |
| --- | --- | --- | --- | --- | --- | --- | --- | --- | --- | --- | --- | --- |
| Alpha | 0.05 |  |  |  |  |  |  |  |  |  |  |  |
|  |  |  |  |  |  |  |  |  |  | N | Mean | Std. Deviation |
| Source of Variation | % of total variation | P value | P value summary | Significant? | |  |  |  |  |  |  |  |
| Interaction | 0.5032 | 0.9951 | ns | No |  |  |  | Hand Eye Coordination0 | 1 | 20 | 56.9 | 12.53 |
| Row Factor | 2.548 | 0.0361 | * | Yes |  |  |  |  | 2 | 19 | 44.37 | 19.41 |
| Column Factor | 8.503 | <0.0001 | **** | Yes |  |  |  |  | 3 | 20 | 50 | 15.44 |
|  |  |  |  |  |  |  |  |  | 4 | 20 | 54.8 | 15.57 |
| ANOVA table | SS (Type III) | DF | MS | F (DFn | DFd) | P value |  |  | Total | 79 | 51.61 | 16.29 |
| Interaction | 490.2 | 9 | 54.47 | F (9 | 300) = 0.1898 | P=0.9951 |  | Hand Eye Coordination10 | 1 | 20 | 59.85 | 12.22 |
| Row Factor | 2482 | 3 | 827.2 | F (3 | 300) = 2.882 | P=0.0361 |  |  | 2 | 19 | 51.84 | 19.32 |
| Column Factor | 8283 | 3 | 2761 | F (3 | 300) = 9.620 | P<0.0001 |  |  | 3 | 20 | 54.25 | 16.32 |
| Residual | 86102 | 300 | 287 |  |  |  |  |  | 4 | 20 | 61.9 | 19.85 |
|  |  |  |  |  |  |  |  |  | Total | 79 | 57.03 | 17.32 |
| Data summary | |  |  |  |  |  |  | Hand Eye Coordination30 | 1 | 20 | 64.35 | 15.18 |
| Number of columns (Column Factor) | 4 |  |  |  |  |  |  |  | 2 | 19 | 48.26 | 13.65 |
| Number of rows (Row Factor) | 4 |  |  |  |  |  |  |  | 3 | 20 | 55.15 | 17.57 |
| Number of values | 316 |  |  |  |  |  |  |  | 4 | 20 | 61.85 | 17.65 |
|  |  |  |  |  |  |  |  |  | Total | 79 | 57.52 | 17.01 |
|  |  |  |  |  |  |  |  | Hand Eye Coordination60 | 1 | 20 | 64.25 | 10.88 |
|  |  |  |  |  |  |  |  |  | 2 | 19 | 52.16 | 17.4 |
|  |  |  |  |  |  |  |  |  | 3 | 20 | 54.35 | 23.15 |
|  |  |  |  |  |  |  |  |  | 4 | 20 | 64.7 | 20.06 |
|  |  |  |  |  |  |  |  |  | Total | 79 | 58.95 | 18.96 |

**Reaction Time**

| Within each row | compare columns (simple effects within rows) | | | | |  |  |  |  |  |  |  |
| --- | --- | --- | --- | --- | --- | --- | --- | --- | --- | --- | --- | --- |
|  |  |  |  |  |  |  |  |  |  |  |  |  |
| Number of families | 4 |  |  |  |  |  |  |  |  |  |  |  |
| Number of comparisons per family | 3 |  |  |  |  |  |  |  |  |  |  |  |
| Alpha | 0.05 |  |  |  |  |  |  |  |  |  |  |  |
|  |  |  |  |  |  |  |  |  |  |  |  |  |
| Dunnett's multiple comparisons test | Predicted (LS) mean diff. | 95.00% CI of diff. | Significant? | Summary | Adjusted P Value | |  |  | groups | N | Mean | Std. Deviation |
|  |  |  |  |  |  |  |  | Reaction Time0 | 1 | 20 | 283.1 | 42.58 |
| 0 min |  |  |  |  |  |  |  |  | 2 | 19 | 292.37 | 28.3 |
| Oral Caffeine vs. Brushing 2 Min | -9.27 | -38.48 to 19.94 | No | ns | 0.7949 |  |  |  | 3 | 20 | 276.95 | 33.58 |
| Oral Caffeine vs. Brushing 3 Min | 6.15 | -23.06 to 35.36 | No | ns | 0.9261 |  |  |  | 4 | 20 | 289.15 | 41.94 |
| Oral Caffeine vs. Brushing 4 Min | -6.05 | -35.26 to 23.16 | No | ns | 0.9293 |  |  |  | Total | 79 | 285.3 | 36.94 |
|  |  |  |  |  |  |  |  | Reaction Time10 | 1 | 20 | 274.7 | 36.92 |
| 10 min |  |  |  |  |  |  |  |  | 2 | 19 | 292.05 | 46.83 |
| Oral Caffeine vs. Brushing 2 Min | -17.35 | -47.32 to 12.62 | No | ns | 0.3816 |  |  |  | 3 | 20 | 271.05 | 46.48 |
| Oral Caffeine vs. Brushing 3 Min | 3.65 | -26.32 to 33.62 | No | ns | 0.9839 |  |  |  | 4 | 20 | 271.55 | 50.17 |
| Oral Caffeine vs. Brushing 4 Min | 3.15 | -26.82 to 33.12 | No | ns | 0.9896 |  |  |  | Total | 79 | 277.15 | 45.29 |
|  |  |  |  |  |  |  |  | Reaction Time30 | 1 | 20 | 259.4 | 29.26 |
| 30 min |  |  |  |  |  |  |  |  | 2 | 19 | 279.95 | 40.98 |
| Oral Caffeine vs. Brushing 2 Min | -20.55 | -49.76 to 8.663 | No | ns | 0.2314 |  |  |  | 3 | 20 | 252 | 35.47 |
| Oral Caffeine vs. Brushing 3 Min | 7.4 | -21.81 to 36.61 | No | ns | 0.8807 |  |  |  | 4 | 20 | 256.35 | 34.64 |
| Oral Caffeine vs. Brushing 4 Min | 3.05 | -26.16 to 32.26 | No | ns | 0.9897 |  |  |  | Total | 79 | 261.7 | 36.18 |
|  |  |  |  |  |  |  |  | Reaction Time60 | 1 | 20 | 256.15 | 31.61 |
| 60 min |  |  |  |  |  |  |  |  | 2 | 19 | 285.95 | 44.65 |
| Oral Caffeine vs. Brushing 2 Min | -29.8 | -59.01 to -0.5867 | Yes | * | 0.0443 |  |  |  | 3 | 20 | 262.25 | 41.14 |
| Oral Caffeine vs. Brushing 3 Min | -6.1 | -35.31 to 23.11 | No | ns | 0.9277 |  |  |  | 4 | 20 | 240.55 | 34.52 |
| Oral Caffeine vs. Brushing 4 Min | 15.6 | -13.61 to 44.81 | No | ns | 0.447 |  |  |  | Total | 79 | 260.91 | 40.88 |
|  |  |  |  |  |  |  |  |  |  |  |  |  |
|  |  |  |  |  |  |  |  |  |  |  |  |  |
| Test details | Predicted (LS) mean 1 | Predicted (LS) mean 2 | Predicted (LS) mean diff. | SE of diff. | N1 | N2 | q | DF |  |  |  |  |
|  |  |  |  |  |  |  |  |  |  |  |  |  |
| 0 min |  |  |  |  |  |  |  |  |  |  |  |  |
| Oral Caffeine vs. Brushing 2 Min | 283.1 | 292.4 | -9.27 | 12.37 | 20 | 20 | 0.7492 | 300 |  |  |  |  |
| Oral Caffeine vs. Brushing 3 Min | 283.1 | 277 | 6.15 | 12.37 | 20 | 20 | 0.4971 | 300 |  |  |  |  |
| Oral Caffeine vs. Brushing 4 Min | 283.1 | 289.2 | -6.05 | 12.37 | 20 | 20 | 0.489 | 300 |  |  |  |  |
|  |  |  |  |  |  |  |  |  |  |  |  |  |
| 10 min |  |  |  |  |  |  |  |  |  |  |  |  |
| Oral Caffeine vs. Brushing 2 Min | 274.7 | 292.1 | -17.35 | 12.69 | 19 | 19 | 1.367 | 300 |  |  |  |  |
| Oral Caffeine vs. Brushing 3 Min | 274.7 | 271.1 | 3.65 | 12.69 | 19 | 19 | 0.2875 | 300 |  |  |  |  |
| Oral Caffeine vs. Brushing 4 Min | 274.7 | 271.6 | 3.15 | 12.69 | 19 | 19 | 0.2481 | 300 |  |  |  |  |
|  |  |  |  |  |  |  |  |  |  |  |  |  |
| 30 min |  |  |  |  |  |  |  |  |  |  |  |  |
| Oral Caffeine vs. Brushing 2 Min | 259.4 | 280 | -20.55 | 12.37 | 20 | 20 | 1.661 | 300 |  |  |  |  |
| Oral Caffeine vs. Brushing 3 Min | 259.4 | 252 | 7.4 | 12.37 | 20 | 20 | 0.5981 | 300 |  |  |  |  |
| Oral Caffeine vs. Brushing 4 Min | 259.4 | 256.4 | 3.05 | 12.37 | 20 | 20 | 0.2465 | 300 |  |  |  |  |
|  |  |  |  |  |  |  |  |  |  |  |  |  |
| 60 min |  |  |  |  |  |  |  |  |  |  |  |  |
| Oral Caffeine vs. Brushing 2 Min | 256.2 | 286 | -29.8 | 12.37 | 20 | 20 | 2.409 | 300 |  |  |  |  |
| Oral Caffeine vs. Brushing 3 Min | 256.2 | 262.3 | -6.1 | 12.37 | 20 | 20 | 0.493 | 300 |  |  |  |  |
| Oral Caffeine vs. Brushing 4 Min | 256.2 | 240.6 | 15.6 | 12.37 | 20 | 20 | 1.261 | 300 |  |  |  |  |

**Short-Term memory**

| Within each row | compare columns (simple effects within rows) | | | | |  |  |  |  |  |  |  |
| --- | --- | --- | --- | --- | --- | --- | --- | --- | --- | --- | --- | --- |
|  |  |  |  |  |  |  |  |  |  |  |  |  |
| Number of families | 4 |  |  |  |  |  |  |  |  |  |  |  |
| Number of comparisons per family | 3 |  |  |  |  |  |  |  |  |  |  |  |
| Alpha | 0.05 |  |  |  |  |  |  |  |  |  |  |  |
|  |  |  |  |  |  |  |  |  |  |  |  |  |
| Dunnett's multiple comparisons test | Predicted (LS) mean diff. | 95.00% CI of diff. | Significant? | Summary | Adjusted P Value | |  |  | groups | N | Mean | Std. Deviation |
|  |  |  |  |  |  |  |  | Short-Term Memory0 | 1 | 20 | 79 | 12.1 |
| 0 min |  |  |  |  |  |  |  |  | 2 | 19 | 73.16 | 10.57 |
| Oral Caffeine vs. Brushing 2 Min | 5.84 | -2.749 to 14.43 | No | ns | 0.2559 |  |  |  | 3 | 20 | 73.5 | 15.99 |
| Oral Caffeine vs. Brushing 3 Min | 5.5 | -3.089 to 14.09 | No | ns | 0.3014 |  |  |  | 4 | 20 | 72.5 | 11.18 |
| Oral Caffeine vs. Brushing 4 Min | 6.5 | -2.089 to 15.09 | No | ns | 0.1818 |  |  |  | Total | 79 | 74.56 | 12.69 |
|  |  |  |  |  |  |  |  | Short-Term Memory10 | 1 | 20 | 85.5 | 9.45 |
| 10 min |  |  |  |  |  |  |  |  | 2 | 19 | 74.47 | 10.39 |
| Oral Caffeine vs. Brushing 2 Min | 11.03 | 2.218 to 19.84 | Yes | ** | 0.0095 |  |  |  | 3 | 20 | 82 | 12.4 |
| Oral Caffeine vs. Brushing 3 Min | 3.5 | -5.312 to 12.31 | No | ns | 0.6685 |  |  |  | 4 | 20 | 79 | 11.65 |
| Oral Caffeine vs. Brushing 4 Min | 6.5 | -2.312 to 15.31 | No | ns | 0.1987 |  |  |  | Total | 79 | 80.32 | 11.56 |
|  |  |  |  |  |  |  |  | Short-Term Memory30 | 1 | 20 | 83.25 | 11.04 |
| 30 min |  |  |  |  |  |  |  |  | 2 | 19 | 78.95 | 12.43 |
| Oral Caffeine vs. Brushing 2 Min | 4.3 | -4.289 to 12.89 | No | ns | 0.4988 |  |  |  | 3 | 20 | 88 | 8.94 |
| Oral Caffeine vs. Brushing 3 Min | -4.75 | -13.34 to 3.839 | No | ns | 0.4187 |  |  |  | 4 | 20 | 90.5 | 11.46 |
| Oral Caffeine vs. Brushing 4 Min | -7.25 | -15.84 to 1.339 | No | ns | 0.1186 |  |  |  | Total | 79 | 85.25 | 11.68 |
|  |  |  |  |  |  |  |  | Short-Term Memory60 | 1 | 20 | 89 | 12.94 |
| 60 min |  |  |  |  |  |  |  |  | 2 | 19 | 81.58 | 12.14 |
| Oral Caffeine vs. Brushing 2 Min | 7.42 | -1.169 to 16.01 | No | ns | 0.107 |  |  |  | 3 | 20 | 86.5 | 8.75 |
| Oral Caffeine vs. Brushing 3 Min | 2.5 | -6.089 to 11.09 | No | ns | 0.8323 |  |  |  | 4 | 20 | 90.5 | 10.5 |
| Oral Caffeine vs. Brushing 4 Min | -1.5 | -10.09 to 7.089 | No | ns | 0.9555 |  |  |  | Total | 79 | 86.96 | 11.48 |
|  |  |  |  |  |  |  |  |  |  |  |  |  |
|  |  |  |  |  |  |  |  |  |  |  |  |  |
| Test details | Predicted (LS) mean 1 | Predicted (LS) mean 2 | Predicted (LS) mean diff. | SE of diff. | N1 | N2 | q | DF |  |  |  |  |
|  |  |  |  |  |  |  |  |  |  |  |  |  |
| 0 min |  |  |  |  |  |  |  |  |  |  |  |  |
| Oral Caffeine vs. Brushing 2 Min | 79 | 73.16 | 5.84 | 3.638 | 20 | 20 | 1.605 | 300 |  |  |  |  |
| Oral Caffeine vs. Brushing 3 Min | 79 | 73.5 | 5.5 | 3.638 | 20 | 20 | 1.512 | 300 |  |  |  |  |
| Oral Caffeine vs. Brushing 4 Min | 79 | 72.5 | 6.5 | 3.638 | 20 | 20 | 1.787 | 300 |  |  |  |  |
|  |  |  |  |  |  |  |  |  |  |  |  |  |
| 10 min |  |  |  |  |  |  |  |  |  |  |  |  |
| Oral Caffeine vs. Brushing 2 Min | 85.5 | 74.47 | 11.03 | 3.732 | 19 | 19 | 2.955 | 300 |  |  |  |  |
| Oral Caffeine vs. Brushing 3 Min | 85.5 | 82 | 3.5 | 3.732 | 19 | 19 | 0.9378 | 300 |  |  |  |  |
| Oral Caffeine vs. Brushing 4 Min | 85.5 | 79 | 6.5 | 3.732 | 19 | 19 | 1.742 | 300 |  |  |  |  |
|  |  |  |  |  |  |  |  |  |  |  |  |  |
| 30 min |  |  |  |  |  |  |  |  |  |  |  |  |
| Oral Caffeine vs. Brushing 2 Min | 83.25 | 78.95 | 4.3 | 3.638 | 20 | 20 | 1.182 | 300 |  |  |  |  |
| Oral Caffeine vs. Brushing 3 Min | 83.25 | 88 | -4.75 | 3.638 | 20 | 20 | 1.306 | 300 |  |  |  |  |
| Oral Caffeine vs. Brushing 4 Min | 83.25 | 90.5 | -7.25 | 3.638 | 20 | 20 | 1.993 | 300 |  |  |  |  |
|  |  |  |  |  |  |  |  |  |  |  |  |  |
| 60 min |  |  |  |  |  |  |  |  |  |  |  |  |
| Oral Caffeine vs. Brushing 2 Min | 89 | 81.58 | 7.42 | 3.638 | 20 | 20 | 2.04 | 300 |  |  |  |  |
| Oral Caffeine vs. Brushing 3 Min | 89 | 86.5 | 2.5 | 3.638 | 20 | 20 | 0.6873 | 300 |  |  |  |  |
| Oral Caffeine vs. Brushing 4 Min | 89 | 90.5 | -1.5 | 3.638 | 20 | 20 | 0.4124 | 300 |  |  |  |  |

**Stroop Effect**

| Within each row | compare columns (simple effects within rows) | | | | |  |  |  |  |  |  |  |  |
| --- | --- | --- | --- | --- | --- | --- | --- | --- | --- | --- | --- | --- | --- |
|  |  |  |  |  |  |  |  |  |  |  |  |  |  |
| Number of families | 4 |  |  |  |  |  |  |  |  |  |  |  |  |
| Number of comparisons per family | 3 |  |  |  |  |  |  |  |  |  |  |  |  |
| Alpha | 0.05 |  |  |  |  |  |  |  |  |  |  |  |  |
|  |  |  |  |  |  |  |  |  |  | groups | N | Mean | Std. Deviation |
| Dunnett's multiple comparisons test | Predicted (LS) mean diff. | 95.00% CI of diff. | Significant? | Summary | Adjusted P Value | |  |  | Stroop Effect0 | 1 | 20 | 1469.35 | 345.21 |
|  |  |  |  |  |  |  |  |  |  | 2 | 19 | 1682.16 | 335.9 |
| 0 min |  |  |  |  |  |  |  |  |  | 3 | 20 | 1522.15 | 278.23 |
| Oral Caffeine vs. Brushing 2 Min | -212.8 | -391.1 to -34.54 | Yes | * | 0.0143 |  |  |  |  | 4 | 20 | 1388.94 | 422.07 |
| Oral Caffeine vs. Brushing 3 Min | -52.8 | -231.1 to 125.5 | No | ns | 0.8253 |  |  |  |  | Total | 79 | 1513.54 | 358.81 |
| Oral Caffeine vs. Brushing 4 Min | 80.41 | -97.86 to 258.7 | No | ns | 0.5793 |  |  |  | Stroop Effect10 | 1 | 20 | 1277.8 | 214.01 |
|  |  |  |  |  |  |  |  |  |  | 2 | 19 | 1383.11 | 147.76 |
| 10 min |  |  |  |  |  |  |  |  |  | 3 | 20 | 1245.45 | 158.52 |
| Oral Caffeine vs. Brushing 2 Min | -105.3 | -288.2 to 77.60 | No | ns | 0.386 |  |  |  |  | 4 | 20 | 1295.35 | 229.42 |
| Oral Caffeine vs. Brushing 3 Min | 32.35 | -150.6 to 215.3 | No | ns | 0.9539 |  |  |  |  | Total | 79 | 1299.38 | 194.2 |
| Oral Caffeine vs. Brushing 4 Min | -17.55 | -200.5 to 165.4 | No | ns | 0.992 |  |  |  | Stroop Effect30 | 1 | 20 | 1223.9 | 230.05 |
|  |  |  |  |  |  |  |  |  |  | 2 | 19 | 1367.53 | 181.36 |
| 30 min |  |  |  |  |  |  |  |  |  | 3 | 20 | 1247.4 | 139.49 |
| Oral Caffeine vs. Brushing 2 Min | -143.6 | -321.9 to 34.64 | No | ns | 0.1439 |  |  |  |  | 4 | 20 | 1200.05 | 200.37 |
| Oral Caffeine vs. Brushing 3 Min | -23.5 | -201.8 to 154.8 | No | ns | 0.9798 |  |  |  |  | Total | 79 | 1258.35 | 197.77 |
| Oral Caffeine vs. Brushing 4 Min | 23.85 | -154.4 to 202.1 | No | ns | 0.9789 |  |  |  | Stroop Effect60 | 1 | 20 | 1177.85 | 217.16 |
|  |  |  |  |  |  |  |  |  |  | 2 | 19 | 1271.53 | 190.01 |
| 60 min |  |  |  |  |  |  |  |  |  | 3 | 20 | 1152.2 | 126.66 |
| Oral Caffeine vs. Brushing 2 Min | -93.68 | -272.0 to 84.59 | No | ns | 0.4601 |  |  |  |  | 4 | 20 | 1154.6 | 173.58 |
| Oral Caffeine vs. Brushing 3 Min | 25.65 | -152.6 to 203.9 | No | ns | 0.9741 |  |  |  |  | Total | 79 | 1188 | 182.78 |
| Oral Caffeine vs. Brushing 4 Min | 23.25 | -155.0 to 201.5 | No | ns | 0.9804 |  |  |  |  |  |  |  |  |
|  |  |  |  |  |  |  |  |  |  |  |  |  |  |
|  |  |  |  |  |  |  |  |  |  |  |  |  |  |
| Test details | Predicted (LS) mean 1 | Predicted (LS) mean 2 | Predicted (LS) mean diff. | SE of diff. | N1 | N2 | q | DF |  |  |  |  |  |
|  |  |  |  |  |  |  |  |  |  |  |  |  |  |
| 0 min |  |  |  |  |  |  |  |  |  |  |  |  |  |
| Oral Caffeine vs. Brushing 2 Min | 1469 | 1682 | -212.8 | 75.5 | 20 | 20 | 2.818 | 300 |  |  |  |  |  |
| Oral Caffeine vs. Brushing 3 Min | 1469 | 1522 | -52.8 | 75.5 | 20 | 20 | 0.6993 | 300 |  |  |  |  |  |
| Oral Caffeine vs. Brushing 4 Min | 1469 | 1389 | 80.41 | 75.5 | 20 | 20 | 1.065 | 300 |  |  |  |  |  |
|  |  |  |  |  |  |  |  |  |  |  |  |  |  |
| 10 min |  |  |  |  |  |  |  |  |  |  |  |  |  |
| Oral Caffeine vs. Brushing 2 Min | 1278 | 1383 | -105.3 | 77.47 | 19 | 19 | 1.359 | 300 |  |  |  |  |  |
| Oral Caffeine vs. Brushing 3 Min | 1278 | 1245 | 32.35 | 77.47 | 19 | 19 | 0.4176 | 300 |  |  |  |  |  |
| Oral Caffeine vs. Brushing 4 Min | 1278 | 1295 | -17.55 | 77.47 | 19 | 19 | 0.2266 | 300 |  |  |  |  |  |
|  |  |  |  |  |  |  |  |  |  |  |  |  |  |
| 30 min |  |  |  |  |  |  |  |  |  |  |  |  |  |
| Oral Caffeine vs. Brushing 2 Min | 1224 | 1368 | -143.6 | 75.5 | 20 | 20 | 1.902 | 300 |  |  |  |  |  |
| Oral Caffeine vs. Brushing 3 Min | 1224 | 1247 | -23.5 | 75.5 | 20 | 20 | 0.3112 | 300 |  |  |  |  |  |
| Oral Caffeine vs. Brushing 4 Min | 1224 | 1200 | 23.85 | 75.5 | 20 | 20 | 0.3159 | 300 |  |  |  |  |  |
|  |  |  |  |  |  |  |  |  |  |  |  |  |  |
| 60 min |  |  |  |  |  |  |  |  |  |  |  |  |  |
| Oral Caffeine vs. Brushing 2 Min | 1178 | 1272 | -93.68 | 75.5 | 20 | 20 | 1.241 | 300 |  |  |  |  |  |
| Oral Caffeine vs. Brushing 3 Min | 1178 | 1152 | 25.65 | 75.5 | 20 | 20 | 0.3397 | 300 |  |  |  |  |  |
| Oral Caffeine vs. Brushing 4 Min | 1178 | 1155 | 23.25 | 75.5 | 20 | 20 | 0.3079 | 300 |  |  |  |  |  |
